# Zebrafish sex determination and differentiation: Involvement of FTZ-F1 genes

**DOI:** 10.1186/1477-7827-3-63

**Published:** 2005-11-10

**Authors:** Jonas von Hofsten, Per-Erik Olsson

**Affiliations:** 1 Department of Molecular Biology, Umeå University, SE-901 87 Umeå, Sweden; 2 Örebro Life Science Center, Department of Natural Science, Örebro University, SE-701 82 Örebro, Sweden

## Abstract

Sex determination is the process deciding the sex of a developing embryo. This is usually determined genetically; however it is a delicate process, which in many cases can be influenced by environmental factors. The mechanisms controlling zebrafish sex determination and differentiation are not known. To date no sex linked genes have been identified in zebrafish and no sex chromosomes have been identified. However, a number of genes, as presented here, have been linked to the process of sex determination or differentiation in zebrafish. The zebrafish FTZ-F1 genes are of central interest as they are involved in regulating interrenal development and thereby steroid biosynthesis, as well as that they show expression patterns congruent with reproductive tissue differentiation and function. Zebrafish can be sex reversed by exposure to estrogens, suggesting that the estrogen levels are crucial during sex differentiation. The Cyp19 gene product aromatase converts testosterone into 17 beta-estradiol, and when inhibited leads to male to female sex reversal. FTZ-F1 genes are strongly linked to steroid biosynthesis and the regulatory region of Cyp19 contains binding sites for FTZ-F1 genes, further linking FTZ-F1 to this process. The role of FTZ-F1 and other candidates for zebrafish sex determination and differentiation is in focus of this review.

## Sex determination

Among mammals sex is usually defined by the presence or absence of the sex specific chromosome Y. In many, but not all, fish species there is also a chromosomal background to sex determination. Several fishes, including most salmonids, have heterogametic males and homogametic females, similar to the mammalian XY/XX-system [1-3]. Other species, such as *Poecilia*, have homogametic males and heterogametic females (ZZ/ZW), which also is the case for birds [4]. Some species of the Poecilid platyfish *Xiphophorus*, utilize a system with three sex chromosomes [5]. In yet other species sex determination is influenced by environmental factors such as the temperature surrounding the developing embryo [6-8]. Hermaphroditism is also a common feature of several fish species. Several studies have shown that species with genetic sex determination can be directed to produce genetically sex-reversed offspring. This is accomplished either by treating the fish with hormones, which can induce sex reversal in synchronous hermaphroditic fish [9,10] and masculinization/feminization in gonochoristic species, or by incubating embryos in certain temperatures or pH [11]. The proportion of males usually increases with temperature whereas lower temperatures favour females. In the case of pH, species differences have been observed.

There are few studies of sex determination in fish and the genetic mechanisms behind sex determination in fish remain largely unknown. Environmental factors, including endocrine disrupters such as diethylstilbestrol, PCBs or dioxins, can affect both teleost and mammalian reproductive systems, but do not seem to alter sex ratios or cause sex reversals in mammals. This indicates that mammalian sex determination is more strictly genetic, and shows less gonad plasticity than teleosts. However, it has been observed that a number of genes, both sex-linked and autosomal, display dosage effects in mammals (Table 1), suggesting that allelic variants could account for differences in gene function.

**Table 1:** Chromosomal location and dose effects. Several genes involved in mammalian sex determination have dose effects leading to sex reversal.

***Chromosomal location***	***Gene***	***Number of copies***	***Phenotype***	***Reference***
Y Chromosome	SRY	0	Female	
		1	Male	[12]
X Chromosome	Dax-1	1	Normal	
		2	Female XY	[13]
Autosomal	Sox9	1	Female XY	[14,15]
Masculinizing		2	Normal	
		3	Male XX	[16]
Autosomal	SF-1	1	Female XY	[17]
Masculinizing		2	Normal	
Autosomal	WT1	1	Female XY	[18]
Masculinizing		2	Normal	
Autosomal	Dmrt1	1	Female XY	[19]
Masculinizing		2	Normal	

While the developmental mechanisms by which the mammalian gonads are formed have been thoroughly studied and several genes involved have been identified, only a few of these genes have been identified in fish. The functions of these genes have not been fully elucidated in fish and both conserved and divergent functions between mammals and fish have been suggested. As zebrafish is an important vertebrate model for developmental biology it is vital that the basic developmental mechanisms of sex determination are further studied in this species. In the present review we discuss the roles of genes involved in sex determination with a focus on the potential role of FTZ-F1 genes in zebrafish sex determination and differentiation. From the present knowledge of these genes in zebrafish we attempt to present a model for zebrafish sex determination and differentiation.

## Formation and differentiation of gonads

There is a close anatomical relationship between the development of the genital ridge and the excretory system during early ontogeny of all vertebrates, including fish. A mesodermal layer ventral to the somites differentiates into structures involved in excretion and reproduction. There are species differences in how closely connected these structures are in regard to sharing ducts for secretion [20]. The teleost gonads are similar to those in mammals. The testis contains Sertoli and Leydig cells in addition to germ cells, and the ovary consists of thecal cells and granulosa cells surrounding the ovum. In both teleosts and mammals the interstitial cells (Leydig and theca), Sertoli cells and granulosa cells are of the same origin. An important difference is that the mammalian gonads are terminally developed into either testis or ovary, while fish gonads often retain the ability to change, making them sequential hermaphrodites [21]. Immature teleost gonads can be directed to develop into testes or ovaries, regardless of chromosomal background, by hormone treatment [22,11]. Far too few fish species have been studied with regard to gonad development to be able to develop a general model of how this occurs.

The zebrafish has become a useful vertebrate model system and is probably the most studied fish in developmental biology. The zebrafish diploid genome consists of 50 chromosomes and no specific sex chromosomes have been identified. The use of synaptonemal complex studies rather indicates that no sex chromosomes exist in zebrafish [23,24]. Teleosts have a partially duplicated genome, which in zebrafish has been determined by studying HOX-clusters [25], and this further complicates the elucidation of potential sex linked genes. In theory, there may be smaller genomic differences that account for or direct the development toward two separate sexes. By studying patterns of inheritance, zebrafish have been suggested to have XY-like chromosomal background [26]. However, the opposite system, with heterogametic females in zebrafish has also been suggested [27]. The inconsistency of results regarding sex chromosomes in zebrafish suggests that the sex determining system is labile and that no clear sex determining chromosome exist. The zebrafish is sexually mature after approximately three months, but separate sexes can be detected after 21–23 days post fertilization (dpf) [26]. Prior to sex differentiation all zebrafish develop ovary-like gonads, regardless of chromosomal background. Ovarian development is the default pathway, which is initiated after 10 dpf and progress until 20 dpf. At 21 dpf until approximately 30 dpf testis development is initiated in males simultaneously with ovarian apoptosis.

The gonad development in zebrafish begins during embryogenesis. Using Vasa as a marker gene, germ cells can be detected in the area ventral to the third to fifth somite at the six-somite stage [28]. While germ cells can be detected earlier, they are not properly positioned until around the 6 somite stage. So far no studies have been made regarding markers for gonadal steroidogenic precursor cells, rendering it difficult to know exactly where these cells are located in the embryo. But, it is likely that these cells derive from an area close to where Vasa is detected at the six-somite stage. The zebrafish Wilms Tumour-1 (WT1) is also initially detected in an area corresponding to that of Vasa but is later expressed in the pronephric ducts [29], making this region probable for the development of the rest of the zebrafish gonadal cells.

## Candidate genes in zebrafish sex determination

In zebrafish, little information exists regarding sex determination and the potential presence of sex chromosomes. To date, no sex-linked genes have been identified. However, a number of genes, as presented here, have been linked to the process of sex determination or differentiation in zebrafish. Since none of the genes have been shown to be sex-linked, it is not likely that any of the below listed genes is the single factor responsible for specifying sex in zebrafish. Still, the expression patterns and regulatory mechanisms of these genes leads to the conclusion that they are part of a signalling network responsible for the development of sex specific gonads. In line with observations on mammals (Table 1) gene dosage effects may be a factor involved in zebrafish sex determination. Since no sex-linked genes have been found in zebrafish, allelic variants and dosage effects of autosomal genes, such as the *Fushi Tarazu *factor-1 (FTZ-F1) genes, SRY HMG box related gene 9 (Sox9), WT-1, Anti-Mullerian Hormone (AMH), doublesex-mab 3 related gene (Dmrt1) and GATA4 (a zinc finger transcription factor) may be involved in determining sex and directing gonad development. The dosage dependent region on X (Dax-1) is highly involved in female sex determination in mammals, but no dax-1 gene homologue has so far been identified in zebrafish. The Dax-1 gene has however been identified in the Nile Tilapia [30], suggesting that other fish species may also have Dax-1 homologues.

## SOX9

Even when sex determination in teleost fish has a genetic background, they lack an equivalent to the testis-determining factor SRY found in mammals. However, several HMG-box containing genes, Sox-genes, have been identified in fish [31-33]. In zebrafish, two Sox9 genes, termed Sox9a and Sox9b, have been identified. Both contain the HMG-box and are able to bind the AACAAAG recognition site in a similar manner as murine Sox9 [34]. The expression patterns of Sox9a and b are dissimilar in adult zebrafish. Sox9a displays a broad expression pattern and has been found in brain, kidney, muscle, testis and pectoral fin, whereas Sox9b is only found in ovary. During embryogenesis Sox9a and b are both expressed in cells involved in craniofacial development and in the brain [34]. In addition Sox9a has been shown to be essential for chondrogenic development [35] and Sox9b has been implicated to be involved in neural crest development [36]. Whether Sox9a and/or b are involved in sex determination or differentiation has so far not been studied. However, an HMG-Box cis element has been identified in gene promoter of *fushi tarazu *factor 1a (ff1a) [37]. Sox9a is also able to specifically bind this site *in vitro *(von Hofsten *et al*., unpublished) indicating that a regulatory connection between Sox9a and ff1a is present in zebrafish. Zebrafish embryos homozygous for jellyfish (jef) (mutations in sox9a) show craniofacial defects and lack of cartilage similar to humans with campomelic dysplasia [35]. The jef strain is still able to reproduce, which leads to the conclusion that Sox9a alone does not direct sex determination and differentiation in zebrafish.

## AMH

AMH may not be excluded as a factor involved in the sex determining process in zebrafish. Although fish lack Müllerian ducts, other AMH functions may be important for gonad development. In mammals AMH is, in addition to Müllerian degeneration, involved in regulation of gonadal steroidogenesis. AMH inhibits the expression of aromatase in developing gonads [38]. It also negatively modulates the differentiation and function of Leydig cells [39] by down regulating several enzymes involved in the steroidogenic pathway. Ovarian cell growth is inhibited by AMH *in vitro *[40]. An AMH related gene, eel spermatogenesis related substances 21 (eSRS21) identified in the Japanese eel is mainly expressed in Sertoli cells and down regulates 11 KT induced spermatogenesis. This indicates that eSRS21 and genes related to AMH have important reproductive functions and are involved in sex determination and differentiation in fish [41]. In zebrafish, we recently cloned an AMH cDNA and observed that it was expressed exclusively in gonads [42]. AMH expression was, by in situ hybridization, found predominantly in Sertoli cells in testis and in the follicular layer in ovaries. Interestingly, AMH is co-expressed both with the Steroidogenic Factor-1 (SF-1) homologue ff1d and Sox9a within these cells [42,43]. AMH displays complex regulation in mammals, involving several factors, including the FTZ-F1 related gene SF-1, GATA4, Sox9 and WT1 [44,45]. The transcriptional regulation of zebrafish AMH has so far not been elucidated. However, the AMH gene promoter sequence contains putative binding sites for the same transcription factors that regulate mammalian AMH, indicating a conserved regulatory mechanism in vertebrates.

## WT1

As in mammals, the anlagen for the excretion and reproductive systems both derive from intermediate mesoderm [46-48]. WT1 was originally found to be a suppressor of Wilms tumour, as individuals with inactivated WT1 developed the Wilms tumour condition [49]. WT1 is also a crucial factor in the differentiation of renal tissue. In zebrafish, WT1 has been shown to be expressed in the intermediate mesoderm prior to and during renal tissue differentiation [29]. It is also essential for the steroidogenic interrenal development together with ff1b [50]. WT1 is thereby an important factor in the early events during development of gonadal primordium.

## FTZ-F1 (NR5A)

The *Drosophila *homeobox gene *fushi tarazu *(ftz) was initially identified as a central factor for segmentation, as inhibition of ftz resulted in the development of fewer segments [51,52]. The *fushi tarazu *factor-1 (FTZ-F1) was later identified as the key regulator of ftz expression [53,54]. Genes homologous to the *Drosophila *FTZ-F1 have subsequently been identified in several species in different phyla [55-63]. Several different names have been given to these homologues, including steroidogenic factor-1 (SF-1), adrenal-4-binding protein (Ad4BP), embryonal long terminal repeat-binding protein (ELP), α-fetoprotein transcription factor (FTF) and liver receptor homologue-1 (LRH-1). However, lately a nomenclature system presented by the nuclear receptor committee groups the FTZ-F1 homologues under the name NR5A [64].

The mammalian genome contains two FTZ-F1 homologues (NR5A1 and NR5A2). NR5A1 contains the SF-1 related genes, which are closely connected to steroidogenesis. In mammals, the NR5A1 genes are expressed in steroidogenic tissues, are key regulators of steroidogenesis and are involved in the testis determining pathway during sex determination [65-67]. The NR5A2 genes are linked to regulation of the estrogen-binding α-fetoprotein [60]. The mammalian NR5A2 genes are expressed in steroidogenic tissues as well as liver, pancreas and intestine, but appear to be more involved in cholesterol metabolism than steroid synthesis or sex determination.

## Zebrafish FTZ-F1

**Figure 1:**
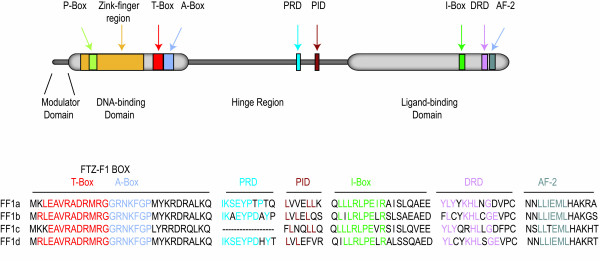
**General structure of zebrafish FTZ-F1 proteins**. The zebrafish FTZ-F1 proteins consist of four main regions, the modulator domain, DNA-binding domain (DBD), hinge region and the ligand-binding domain. The DBD contains a Zink-finger region, an A- and P-box for recognition of the FTZ-F1 response element, and a T-box for stabilising the DNA-binding. The proximal repressive- and interactive domains (PRD and PID) are used for interactions with co-repressors and co-activators. The ligand binding-domain containing the I-box and AF-2 region, which both are involved in ligand binding and transactivation, and a distal repressive domain (DRD) for co-repressor binding.

**Figure 2:**
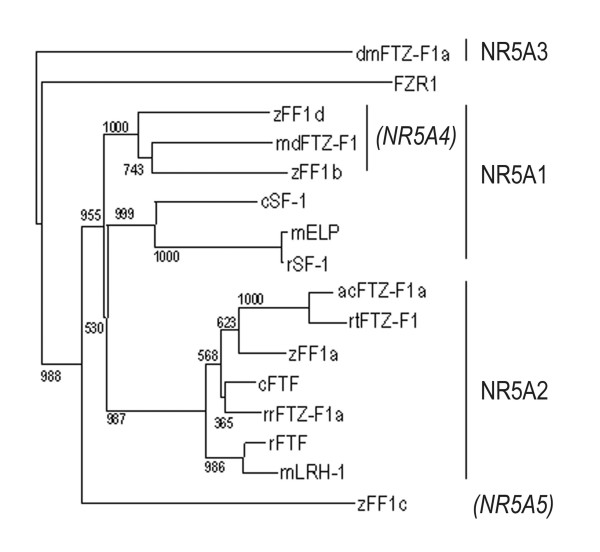
**NR5A sequence similarity analysis displayed in a radial tree**. Clades containing subgroups NR5A1, NR5A2, NR5A3 and NR5A4 are indicated. Arctic char FF1a (acFF1a); Mouse LRH-1 (mLRH-1); Rat SF-1 (rSF-1); Mouse ELP (mELP); *Rana rugosa *FTZ-F1 (rrFTZ-F1); Zebrafish ff1b (zff1b); Zebrafish ff1a (zff1a); Zebrafish ff1c (zff1c); Zebrafish ff1d (zff1d); Rat FTF (rFTF); Medaka FTZ-F1 (mFTZ-F1); Rainbow trout FTZ-F1 (rtFTZ-F1); Chick SF-1 (cSF-1); Chick FTF (cFTF) and *Drosophila melanogaster *ftz-f1 (dmFTZ-F1). Modified from [42].

**Figure 3:**
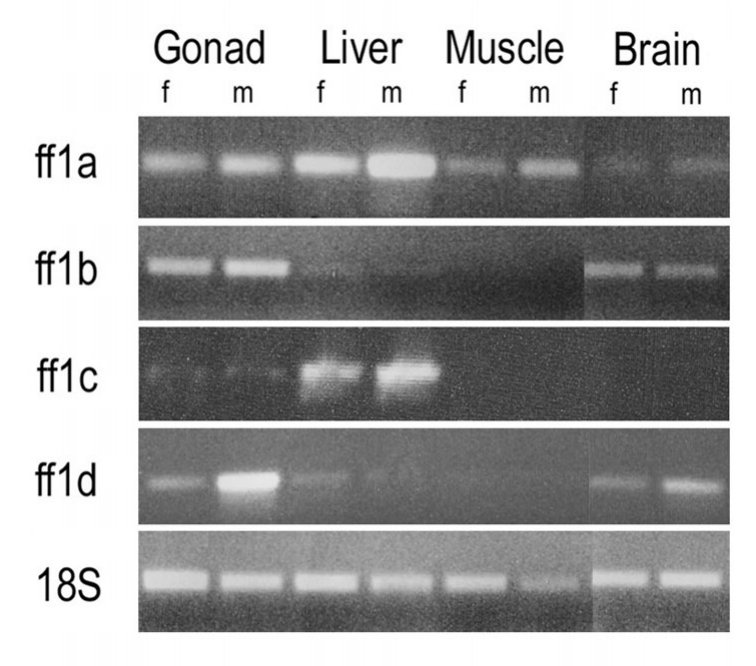
**Tissue distribution of ff1a, ff1b, ff1c and ff1d in adult zebrafish, detected by RT-PCR**. The four ff1 genes show differential expression with the ff1a gene being expressed in most tissues with high expression in liver. The ff1b and ff1d genes are both expressed in gonads and brain with the ff1d showing higher expression in testis than in ovary. The ff1c gene is primarily expressed in the liver. m: male tissue, f: female tissue. Modified from [42].

FTZ-F1 homologues have been identified in a number of teleost species [55-57,68-70]. Several teleosts have multiple variants of FTZ-F1 genes. The roles and functions of these genes are not completely elucidated, but all studies conducted so far indicate an involvement in the reproductive axis or in steroidogenesis. The ff1 proteins share the general structure of other nuclear receptors. They contain a DNA binding domain (DBD) with two Zn-fingers and the FTZ-F1 box for DNA interaction and recognition, a hinge domain that connects to the ligand-binding domain (LBD), which contains the I-box for protein-protein interaction and the activator function-2 (AF-2) domain for transcriptional activation (Fig. 1). Zebrafish is the most extensively studied teleost and four FTZ-F1 genes have been identified (ff1a, b, c and d). The arrangement of FTZ-F1 genes into the nuclear receptor 5A subgroups is a suitable system for genes in higher vertebrates, as no indistinguishable genes have been described so far in these animals. However, teleosts and particularly zebrafish are different in more than one way compared to higher vertebrates. Zebrafish have four different FTZ-F1 genes, whereas mammals and higher vertebrates only possess two. The zebrafish genes are not easily arranged within the NR5A subgroups. Zebrafish ff1a and Arctic char ff1 aligns well within the NR5A2 subgroup (Fig. 2), but their expression patterns and suggested functions do not fit the description of the mammalian NR5A2 genes. The zebrafish ff1c does not align well with any of the described subgroups, which further raises the question of how appropriate the subdivisions really are for teleost FTZ-F1. Ff1d and ff1b are similar and aligns together with medaka FTZ-F1 in a subgroup within the NR5A1 clade. This subgroup has previously been named NR5A4, but recent data indicates that the genes in the NR5A4 subgroup are NR5A1 homologous. Recently it was suggested that ff1b and ff1d are of the same origin and arose from ancestral gene duplication [71]. This was supported by the overlapping expression patterns found in embryonic interrenal and pituitary cells [42]. The tissue distribution of ff1b and ff1d is identical, while it differs from ff1a and ff1c (Fig. 3). The combined information of ff1b and ff1d expression patterns, function and sequence similarities to other genes in the NR5A1 group suggest that these genes should be considered as homologues.

### Ff1a

The first FTZ-F1 gene described in zebrafish was ff1a [55]. The gene was named zff1, as no other zebrafish ff1 genes were known at that time. With the identification of additional ff1 genes it was later renamed ff1a. The gene possesses two splicing variants, now designated ff1a-A and ff1a-B. Ff1a-A was, in synergy with ER, shown to transcriptionally activate a gonadotropin promoter, whereas ff1a-B acted as an inhibitor of ff1a-A due to its lack of the AF-2 trans-activation domain. The expression of zebrafish ff1a was later shown to be driven by two separate gene promoters, giving rise to a total of four separate gene transcripts, ff1a-IA, ff1a-IB, ff1a-IIA and ff1aIIB [72].

The functional difference between ff1a proteins consisting of exon one, transcribed via promoter I, and proteins transcribed from promoter II has so far not been studied. However, they differ slightly in their tissue distribution, where promoter I derived transcripts are lacking in brain and heart [72]. The two ff1a promoters contain different putative response elements [37,72]. This suggests that the two promoters regulate ff1a in tissue specific manners and during different developmental stages rather than rendering them separate functions. Also mouse and rat have two separately regulated NR5A promoters [65,73].

Two of the putative response elements in promoter I indicate an involvement in somitogenesis. MyoD and Snail are both transcription factors shown to be involved in somite development [74,75], indicating that promoter I may drive the ff1a expression during somitogenesis. The ff1a IIA gene product is involved in muscle differentiation during somitogenesis. Microinjection of ff1a AII mRNA into the *ubo*-mutant strain, which lack slow-twitch muscle cells, results in partially restored myofibers [76]. Promoter II contains an HMG-Box response element 24–31 bp up stream of the transcription start. An identical response element has been shown to bind Sox9a *in vitro *[34] and Sox9 is hence a putative regulator of gonadal expression of ff1a. Expression of ff1a can be detected in the uro-genital and pronephric duct region during early somitogenesis [77] and by linking the ff1a dual promoter to GFP, early gonadal expression can be detected 5 dpf after microinjection [37]. This indicates a role in early gonad development and differentiation. The Arctic char ff1 homologue has been linked to steroidogenesis by showing a cyclic expression pattern during the reproductive maturation process and 17β-estradiol mediated down-regulation of testicular expression [56]. The phylogenetic relationship indicates that the Arctic char ff1 belongs to the ff1a related genes and should be named ff1a (Fig. 2). Furthermore, the developmental expression pattern of Arctic char ff1a is similar to zebrafish ff1a [78], indicating that teleost ff1a homologues are involved both in steroidogenesis and gonad development.

### Ff1b

The ff1b gene was initially assigned to functions related to pancreatic development as it was co-expressed with pancreas duodenum homeobox-1 (pdx-1) and proinsulin [56]. However, more recent publications suggest that ff1b is an important factor for steroidogenic cell development and that ff1b is required for the differentiation of the interrenal organ [79,80]. The expression of ff1b precedes that of cyp11A and 3βHSD in the embryonic interrenal cells and ff1b morpholino knock down experiments abolishes the expression of these two genes [79].

The down stream transcriptional activation function of ff1b is modulated by protein-protein interactions with homeodomain protein Prox1 [80]. Two domains are needed for the interaction, the I-box and the AF-2 domain, both situated in the LBD. Binding to Prox1 leads to a repression of down stream trans-activation. There is also a co-localization of ff1b and Prox1 expression in the developing interrenal. Due to the conserved I-boxes and AF-2 domains, both ff1a and ff1c are probably able to interact with Prox1, although less efficiently than ff1b.

### Ff1c

There is little information available regarding ff1c functions, regulation or expression patterns. Except for a weak interaction between Prox1 and ff1c presented in Liu *et al*. [80], the sequence published on GenBank, (AF327373) is the only published data available so far. Expression of ff1c can be detected in numerous tissues in adult zebrafish and its highest expression is found in liver and intestine, indicating a role in cholesterol metabolism, similar to ff1a (see Fig. 3). No specific ff1c expression domains have been identified during embryogenesis. Both ff1c and ff1d are similar to ff1a and b in their DNA-binding domains where the FTZ-F1 box is situated and in the ligand binding domains, but are less conserved in their hinge regions. All zebrafish ff1 have highly conserved AF-2 domains and I-Boxes in their LBD.

### Ff1d

Ff1b and ff1d display an overlapping expression pattern during embryogenesis. They share protein domains important for co-factor interactions and have been suggested to be the result of an ancestral gene duplication [42,71]. Even though the two zebrafish NR5A1 genes are similar in several aspects, the shared sequence identities are 62%, which suggests that functional differences are likely to exist. Expression of ff1d in adult zebrafish is restricted to brain, gonads and liver [42]. There also seem to be sexual differences, as ff1d expression is higher in testis than in ovary. In the testis ff1d is highly expressed in interstitial Leydig cells and Sertoli cells, but cannot be detected in germ cells. In ovary ff1d is located to the follicular layer and inside the oocyte [42]. Although the function and regulatory mechanisms of ff1d in these cells needs to be further studied, a possible target of ff1d is AMH, which is co-expressed with ff1d in Sertoli cells and in the follicular layer. During mammalian sex determination and differentiation, the ff1d homolog SF-1 regulates the expression of AMH, leading to the development of male sex characteristics. Therefore it is intriguing that this may be a conserved vertebrate developmental mechanism.

## Dmrt1

The lack of testis-determining factor similar to SRY in fish is not a unique phenomenon. This is also the case for many other lower vertebrates. Genes containing a DM-domain (Dmrt1) have however been identified in fish. DM-domain containing genes are involved in sex determination in both vertebrates and invertebrates [81], which is a unique conservation of function between phyla, not seen in any other gene involved in sex determination. Different Dmrt1 homologues have been shown to be involved in gonad development [82,83] and somitogenesis [84].

The teleost Japanese medaka has specific sex chromosomes (XX/XY), where the DM-domain gene DMY has been mapped to the Y-chromosome and has been shown to be essential for testis differentiation [85,86]. This was determined by isolating the sex-determining region on the male specific Y-chromosome. Except for the DMY-containing region, the X and Y-chromosomes are very similar. This indicates that the medaka Y-chromosome and DMY are, in an evolutionary prospective, relatively new. This theory was later confirmed and DMY was discarded as a universal teleost sex-determining gene, as it was shown to be a species-specific sex-determining strategy [87]. No target genes for DMY have been identified and DMY function in testis development remains unresolved. Treating birds with the aromatase inhibitor fadrozole lead to elevated Dmrt1 levels indicating that Dmrt1 may be down regulated by aromatase [88]. This indicates that Dmrt1 may have an important role in testis determination in teleosts, since alteration of aromatase levels during sex differentiation can cause sex reversals. The regulation of Dmrt1 related genes in teleosts remains unknown, but testicular expression of Dmrt1 is in mammals regulated by GATA4 [89].

## GATA

GATA factors are divided into two families based on expression patterns, structure, and function [90]. GATA-1/2/3 is most commonly associated with haematopoietic cell and neuronal development [91,92]. GATA-4/5/6 are usually linked to organ development, including the urogenital system [93,94]. GATA factors recognize and bind the DNA consensus motif, WGATAR, and closely related sequences. GATA-4 plays an important role as transcriptional regulator of SRY and AMH during mammalian sex determination and differentiation [95]. Studies of zebrafish GATA factors have so far been associated with the development of organ systems other than the urogenital, but binding sites for GATA4 have been found in the cyp19 gene promoter [96,97] suggesting a role in regulating aromatase expression.

## Aromatase

Steroidogenesis, sex determination and differentiation are closely related to each other. SF-1 is one of the crucial factors essential for steroid biosynthesis as well as sex determination and differentiation in mammals. The terminal sex-hormone products in the steroid biosynthesis pathway are androgens and estrogens, and the balance between them leads to the development of proper sex characteristics. Aromatase (Cyp 19) is the product of the cyp19 gene, and is an important regulator of this balance. Aromatase is produced in the gonads and directs the conversion of testosterone into 17β-estradiol.

Like many other fish species, the zebrafish genome contain two aromatase genes designated cyp19a and cyp19b [98]. Cyp19a is highly expressed in the steroidogenic Theca and granulosa cell layer surrounding the oocytes in the ovary, whereas cyp19b is mainly expressed in brain. Thus, while one aromatase gene appears to be involved in gonadal development the other gene may be involved in neuronal development. However, both genes contribute to the regulation of estrogenic responses and may thus influence sex differentiation. The regulation of teleost cyp19 transcription is not completely elucidated, but the zebrafish cyp19a promoter region contains binding sites for Ftz-F1, which suggests a role for ff1 genes in the regulation of cyp19a expression in gonadal tissue [97,99]. Ftz-F1 dependent cyp19 transcription has also been documented in species from turtles to humans [100-102], indicating that this mechanism is conserved in all vertebrates. In many vertebrates, reptiles in particular, the level or activity of aromatase is the deciding factor for sex during development [103,104]. The temperature surrounding the developing embryos influences the activity of aromatase leading to variations in sex ratio [105]. A similar scenario has been documented for several fish species, including zebrafish, suggesting that aromatase is an important factor in sex determination and differentiation in fish. By using an aromatase inhibitor, or by increasing water temperature to 35°C–37°C, oocyte apoptosis can be induced in zebrafish [106]. However, during normal breeding conditions the temperature is not an important factor deciding sex ratios in zebrafish. The role of aromatase remains important in zebrafish sex determination as exposure to the aromatase inhibitor fadrozole results in sex-reversion of female zebrafish [106].

## Sex determination and differentiation pathway

**Figure 4:**
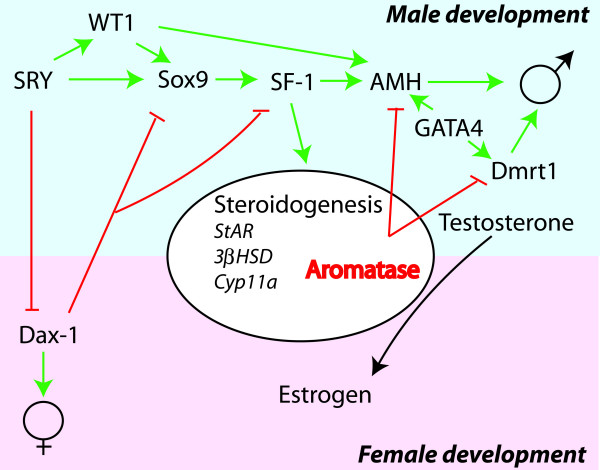
**Involvement of a hierarchy of genes in mammalian sex determination and differentiation**. In XY/XX systems where SRY is the key regulator of sex determination its absence leads to activation of Dax1 and female development. The presence of SRY results in a hierarchy of activation of genes leading to the development of testis. In this hierarchy SF-1 (FTZ-F1) is a key regulator of steroidogenesis and AMH, demonstrating its central role in sex determination and differentiation. ⊥: Inhibition, ↓: stimulation.

Studies of mammals have shown that genes involved in sex determination have multiple functions but that the presence of the SRY gene in animals with XY/XX chromosomal systems leads to male development. As discussed earlier in this review many of the genes involved in mammalian sex determination show dosage dependent effects on sex determination and differentiation. A generalised genetic regulatory pathway can be extracted from the studies conducted on different vertebrate species (Fig. 4). Most identified genes have a primary function in male development. The regulatory pathway includes, and is based on, the presence of SRY and Dax-1, which in an antagonistic manner direct the development of male and female phenotypes in mammals. Lack of the SRY gene, as is the case in teleosts, suggest that when chromosomal sex determination exist it must be regulated by alternative genes. In medaka the Dmrt1 gene was found to be the switch. However, this role for Dmrt1 is specific for medaka and it remains that any of the other known genes in the sex determination cascade could potentially develop into the genetic switch in other teleost species. Or, there may be yet unidentified genes and pathways that participate in teleost sex determination.

## Proposed model

**Figure 5:**
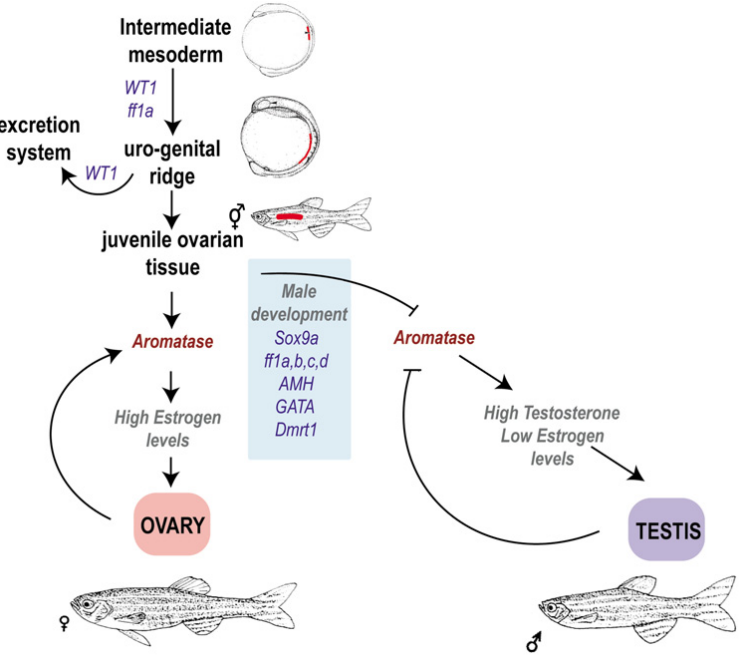
**A generalized model of the involvement of different genes in zebrafish sex determination and differentiation**. While little is known of the hierarchy of genes involved in zebrafish sex determination and differentiation several genes have been identified. While aromatase has been shown to play a central role in zebrafish sex differentiation the environmental and/or genetic mechanisms have not been fully elucidated. ⊥: Inhibition, ↓: stimulation.

From the information obtained to date it is not possible to define a hierarchy of regulation during sex determination in teleosts. Therefore, the proposed model for zebrafish sex determination is based on the same group of male determining genes, excluding SRY and Dax-1, found in mammals while the interplay between the genes remains undefined (Fig. 5). Theoretically, any one of the described genes may become the sex determining gene, as several of them have been shown to cause sex reversals in mammalian model systems in a dosage dependent way (see table 1). The male determining switch may also be dependent on combinatory effects of allelic variants among the genes involved. Furthermore, the regulation of aromatase appears to be crucial for zebrafish sex determination.

The model suggests that ff1a and WT1 are important for the differentiation of the uro-genital tissue, which subsequently develops into renal and gonadal tissue. WT1 is essential for the differentiation of pronephros, and a battery of genes, including the FTZ-F1 genes, Sox9a, GATA4, Dmrt1 and AMH, are involved in the differentiation of gonads. During the critical time period around 25 dpf this battery of genes may direct the development towards male gonads in individuals with the allelic combinations predestined to become male. This would lead to a decrease or absence of aromatase and subsequent reduced estrogen levels and activity, resulting in the onset of ovarian apoptosis, the differentiation of testicular Sertoli cells and increased testosterone levels. The model is based on the observation that adult female zebrafish can be sex-reversed by inhibiting aromatase [106]. This suggests that zebrafish has a high plasticity in their mechanism of gender development and that steroidogenesis plays an essential part in the sex determining process. The complex mechanism of sex determination and differentiation is still far from elucidated and the biochemistry behind it must be further studied to establish protein interactions controlling it. The FTZ-F1 genes are important, as they are involved in the early development of uro-genital tissue and as regulators of steroidogenic cells and their gene expression.
